# 6-Hydroxyflavone and Derivatives Exhibit Potent Anti-Inflammatory Activity among Mono-, Di- and Polyhydroxylated Flavones in Kidney Mesangial Cells

**DOI:** 10.1371/journal.pone.0116409

**Published:** 2015-03-19

**Authors:** Xing Wang, Zhiwei Wang, Preetpal Singh Sidhu, Umesh R. Desai, Qibing Zhou

**Affiliations:** 1 Department of Nanomedicine and Biopharmaceuticals, College of Life Science and Technology, Huazhong University of Science and Technology, Wuhan, Hubei, China; 2 Department of Medicinal Chemistry and Institute for Structural Biology and Drug Discovery, Virginia Commonwealth University, Richmond, Virginia, United States of America; Duke University Medical Center, UNITED STATES

## Abstract

Inflammatory responses by kidney mesangial cells play a critical role in the glomerulonephritis. The anti-inflammatory potential of nineteen mono-, di- and polyhydroxylated flavones including fisetin, quercetin, morin, tricetin, gossypetin, apigenin and myricetin were investigated on rat mesangial cells with lipopolysaccharide (LPS) as the inflammatory stimuli. 6-Hydroxyflavone and 4′,6-dihydroxyflavone exhibited high activity with IC_50_ in the range of 2.0 μM, a much better inhibition potential in comparison to the well-studied polyhydroxylated flavones. Interestingly, the anti-inflammatory activity was not due to direct quenching of NO radicals. Investigation on derivatives with methylation, acetylation or sulfation of 6-hydroxyl group revealed that 6-methoxyflavone was the most potent with an IC_50_ of 192 nM. Mechanistic study indicated that the anti-inflammatory activity of 6-methoxyflavone arose via the inhibition of LPS-induced downstream inducible NO synthase in mesangial cells. The identification of 6-hydroxyflavone and 6-methoxyflavone with potent anti-inflammatory activity in kidney mesangial cells provides a new flavone scaffold and direction to develop naturally derived products for potential nephritis prevention and treatment.

## Introduction

Hydroxylated flavones and derivatives are a group of naturally derived products that exhibit a broad spectrum of anti-inflammatory, anticancer, anti-oxidant and antimicrobial activities [1,2]. Several well-known examples with anti-inflammatory activity include quercetin, morin and apigenin [2,3]. Although there are many studies of flavones and flavonoids on the biological activity in macrophages and cancer cells [1,4,5], the structure-activity relationship study of natural hydroxylated flavones on the renal protection, especially anti-inflammatory activity in kidney mesangial cells has not been reported.

Inflammation in the kidneys due to infection and autoimmune diseases is one of the major causes of glomerulonephritis that leads to deteriorated renal functions resulting in proteinuria and hematuria [6,7]. In glomerulus, kidney mesangial cells are responsible for inflammatory cytokine and oxidative stress production with phagocytic property upon pathological stimuli or injury as well as contraction and relaxation with smooth muscle cell-like property [8]. The biological responses of mesangial cells also play a critical role in glomerulosclerosis in diabetic patients [8]. Recently, several polyhydroxylated flavones have been reported to exhibit protective roles via inhibition of inflammatory pathways in the kidneys. For example, mericetin, a hexahydroxylated flavone has been shown to restore multiple altered renal functions and reduce glomerulosclerosis in the strepozotocin-induced diabetic rat model [9]. In addition, quercetin, the naturally abundant flavonol in fruits and leaves significantly protects the kidneys from Pb (II)-induced toxicity such as oxidative stress and elevated inflammation [10]. Furthermore, hydroxylated flavone luteolin is also a nephraprotective agent to reduce anticancer drug cisplatinum-induced inflammatory responses including activated NF-κB pathway and elevated TNF-α level [11].

In this study, we investigated the potential of anti-inflammatory activity of nineteen mono-, di- and polyhydroxylated flavones on rat mesangial cells with lipopolysaccharide (LPS) as the inflammatory stimuli. Interestingly, we found that 6-hydroxyflavone and 4′,6-dihydroxyflavone exhibited high activity as a unique hydroxylated flavone scaffold rather than the polyhydroxylated flavones. The direct quenching of NO radicals by hydroxylated flavones was then assessed via the inhibition of spontaneous NO formation from sodium nitroprusside in solution. Three derivatives of 6-hydroxyflavone including 6-methoxyflavone, 6-acetoxyflavone and flavone 6-sulfate were also investigated for further structure-activity relationship. The potential molecular targets were finally evaluated by western blot analysis on the LPS-stimulated NF-κB pathway and the downstream inducible NO synthase (iNOS) in kidney mesangial cells.

## Materials and Methods

### Chemicals and Reagents

3-Hydroxyflavone, 6-hydroxyflavone, 7-hydroxyflavone, 6-methoxyflavon, diadzein and resveratrol were obtained from Sigma-Aldrich, USA with a purity of >98%. Di-hydroxylated and polyhydroxylated flavones were obtained from Indofine Chemical Co. (Hillsborough, NJ, USA) with a purity of >98%. Lipopolysaccharide (LPS) from *Escherichia coli* was obtained from Sigma-Aldrich, USA. All other chemicals were obtained from Sinopharm Chemical Reagent Co., Ltd (Shanghai, China) or Sigma Aldrich, USA unless otherwise specified. Stock solutions of all flavonoid compounds were prepared in DMSO. All of the experiments were independently repeated at least three times. NMR spectra were recorded with Bruker Avance-400 NMR spectrometer (Madison, WI, USA). Electrospray ionization mass spectroscopy (ESI-MS) analysis was carried out with a Thermo Fisher TSQ Quantum Max Triple Stage Quadrupole mass spectrometer (MA, USA).

### Cells

Rat mesangial HBZY-1 cells were obtained from China Center for Type Culture Collection (Wuhan, China). Cells were maintained in high glucose DMEM medium (Invitrogen, USA) supplemented with 10% heat-inactivated fetal bovine serum (FBS), 25 mM HEPES, 2 mM L-glutamine, 0.1 mM nonessential amino acids, 1.0 mM sodium pyruvate, 50 U/mL penicillin, and 50 μg/mL streptomycin at 37°C and 5% CO_2_. The cell passage number used for the following investigation was between 10 to 20 passages.

### Total nitrite assay

Mesangial cells were plated on 48-well plates at a density of 50,000 cells per well overnight in DMEM media containing 2% FBS. Cells were treated by flavonoid compounds at a series of concentrations from 0, 0.05, 0.1, 0.5, 1, 5, 10 to 50 μM or 0, 1, 5, 10, 50, 100 to 200 μM based on the initial activity range with or without 10 ng/mL LPS. Total nitrite production in the treatment media after 48 h was measured with Griess assay. Briefly, 100 μL media was mixed with 25 μL of reagent І (1% sulfanilamide in 5% phosphoric acid) and then 25 μL of reagent II (0.1% *N*-1-napthylethylenediamine dihydrochloride in water). The optical absorbance of the resulting solution was recorded at 540 nm with a 318C-microplate reader (Sanco Instrument Ltd. Shanghai, China) after 20 min. The total nitrite concentration per 100 μL treatment media was calculated based on the result from the standard curve with sodium nitrite using the GraphPad Prism software (GraphPad Software, version 5.0, USA). IC_50_ values were obtained using the dose-response analysis provided by the GraphPad Prism software.

### MTT cell viability assay

Cells were plated overnight in growth media (5,000 cells per well) and then treated with each compound for 48 h at increasing concentrations with total DMSO at 0.1%. MTT assay was carried out as reported [12], and cell viability was plotted with GraphPad Prism program.

### NO radical quenching study

Spontaneous generation of NO in solutions was achieved by a freshly prepared sodium nitroprusside stock solution in water (50 mM). The level of total nitrile production was determined by the Griess assay with the standard curve of sodium nitrile as described above. For the assay of NO inhibition by flavonoids, sodium nitroprusside solution (10 mM, 25 μL) was mixed with an equal volume of flavonoid solution in 50% DMSO, and the resulting solution was incubated at room temperature for 10 min. Griess reagent І and Griess reagent II (75 μL each) were subsequently added with a gap of 10 min. The optical density at 540 nm was recorded on a 318C-microplate reader after 15 min. The resulting data were processed and analyzed by the GraphPad Prism Software as described above.

### Synthesis of 6-acetoxyflavone and 6-flavone sulfate sodium salt

6-Acetoxyflavone (**10)** was synthesized from direct acetylation of 6-hydroxyflavone with acetylchloride at 100 mg scale as reported [13] and confirmed by ^1^H NMR analysis. ^1^H NMR (CD_3_Cl): 7.92–7.87 (m, 3H, 3CH), 7.59 (d, ^*3*^ *J* = 8.9 Hz, 1H, CH), 7.55–7.50 (m, 3H, 3CH), 7.44 (dd, ^*3*^ *J* = 8.8 Hz, ^*4*^ *J* = 3.2 Hz, 1H, CH), 6.81 (s, 1H, CH), 2.34 (s, 3H, CH_3_).

6-Flavone sulfate (**11**) was synthesized based on the reported procedure with modifications [14]. Triethylamine (0.30 ml, 2.3 mmol, 11 equiv) and trimethylamine-sulfur trioxide complex (350 mg, 2.1 mmol, 10.0 equiv) were added to a solution of 6-hydroxyflavone (50 mg, 0.21 mmol, 1 equiv) in acetonitrile (0.8 mL). The mixture was exposed to microwaves (50 W, 30 min, 100°C). The resulting solution was concentrated in vacuum, and the residue was dissolved in 2 ml of water. The mixture was loaded onto a Sephadex G-10 column (pre-conditioned with NaCl) and was chromatographed with 20% EtOH/water as the eluent. Appropriate fractions were pooled based on the thin layer chromatograph analysis (15% MeOH in CH_2_Cl_2_) and lyophilized to give 6-flavone sulfate sodium salt (61 mg, 86%). ^1^H NMR (CD_3_OD): 8.00 (d, ^*4*^ *J* = 2.6 Hz, 1H, CH), 7.98–7.95 (m, 2H, 2CH), 7.70 (dd, ^*3*^ *J* = 8.8 Hz, ^*4*^ *J* = 2.8 Hz, 1H, CH), 7.65 (d, ^*3*^ *J* = 8.6 Hz, 1H, CH), 7.52–7.50 (m, 3H, 3CH), 6.82 (s, 1H, CH); ^13^C NMR (CD_3_OD): 180.2, 165.9, 154.7, 151.6, 133.1, 132.7, 130.3, 129.8, 127.6, 125.2, 120.6, 117.5, 107.2. MS (ESI): calc for C_15_H_9_O_6_S (M^-^) 317.01, observed 317.01 (100%); calc for C_15_H_9_O_3_ ([M-SO_3_]^-^) 237.06, observed 237.06 (34%).

### Western blot analysis

Cells were plated in growth media overnight at a density of 1×10^6^ cells per well on a 6-well plate. Cells were first incubated with 6-methoxyflavone or resveratrol for 12 h and then LPS was added to a final concentration of 500 ng/mL. For the western blot analysis, cells were washed with PBS and then lysed with 150 μL RIPA buffer containing protease and phosphatase inhibitor cocktail. The supernatants were collected by centrifuge at 14,000 g×10 min at 4°C and stored at -80°C. The total protein content of lysates was determined by enhanced BCA protein assay kit (Beyotime Institute of Biotechnology, Shanghai, China). Electrophoresis was carried out on a NuPAGE Novex Bis-Tris 4–14% gel (Invitrogen, USA) under the reduced condition. After electrophoresis separation, proteins were transferred to membranes and incubated with rabbit monoclonal phospho-NF-κB-p65 antibody (3033S, 1:1000 dilution, Cell Signaling, USA), rabbit polyclonal iNOS antibody (ab3523, 1:500 dilution, Abcam, USA) or mouse monoclonal β-actin antibody (ANT009, 1:1000 dilution AntGene, China). Targeted proteins were then visualized with Qdot 625 conjugate kit (Invitrogen, USA). Gel images were captured with ZF-258 Gel Imaging System (Shanghai Jiapeng Scientific Co. Ltd, China) under illuminating light at 350 nm.

## Results and Discussion

### Anti-inflammatory activity of mono- and polyhydroxylated flavones in kidney mesangial cells

**Fig 1:**
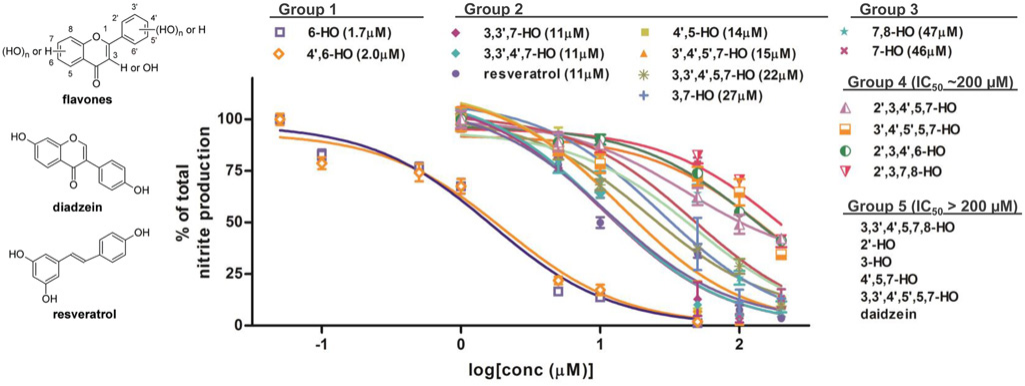
Anti-inflammatory activity of mono-, di- and polyhydroxylated flavones on the LPS-induced NO production in rat kidney mesangial cells. All of the hydroxylated flavones were abbreviated as the positions of hydroxyl groups followed by—HO. The total production of nitrite in the treatment media was measured with Griess assay at 48 h post 10 ng/mL LPS stimulation. The nitrile level in the LPS treatment alone was used as 100%, and IC_50_ values were obtained using the dose-response analysis provided by the Graphpad Prism software as shown in parenthesis. Compounds of group 5 with IC_50_ >200 μM were not shown in the figure.

A total of nineteen hydroxylated flavones including four monohydroxylated, four dihydroxylated, two trihydroxylated, four tetrahydroxylated, three pentahydroxylated and two hexahydroxylated flavones as representatives were screened for the potential anti-inflammatory activity on the LPS-induced NO production in kidney mesangial cells (Fig. 1). This compound list included several well reported bio-active hydroxylated flavones such as fisetin [15], quercetin [4,5], morin [2], tricetin [15], gossypetin [16], apigenin [2,4] and myricetin [2], which exhibit broad spectrum anti-inflammatory and anticancer effects.

The inhibitory activity of the LPS-induced NO production in kidney cells by hydroxylated flavones in our study could be categorized into five groups with IC_50_ in the range of 1.7 μM to > 200 μM (Fig. 1). Because high activities have been found with Group one and two hydroxylated flavones such as 6-hydroxyflavone and 4′,6-dihydroxyflavone, the activity of hydroxylated flavones was not assessed above 200 μM. In general, the inhibitory activity of the mono- and dihydroxyflavones were found across the whole range while tri-, tetra- and pentahydroxyflavones exhibited modest to low activity in the Group two, four and five. Surprisingly, the most polyhydroxylated flavones, i.e., 3,3′,4′,5,7,8-hexahydroxyflavone (gossypetin) and 3,3′,4′,5,5′,7-hexahydroxyflavone (myricetin) did not showed any significant inhibition of the LPS-induced NO production below 200 μM. On the other hand, resveratrol, a well-known anti-inflammatory natural product [2] as the positive control exhibited a relative potent inhibitory effect with an IC_50_ of 11 μM, similar to the group two hydroxylated flavones including 3,3′,7-trihydroxy- and 3,3′,4′,7-tetrahydroxyflavones (Fig. 1). In addition, minimal or weak cytotoxicity was only observed after 48 h with several hydroxylated flavones at concentrations much higher than those of IC_50_ against LPS-induced NO production, typically by 5 fold for Group one and Group two hydroxylated flavones (see S1–3 Tables). These results implied that the anti-inflammatory activity of hydroxylated flavones was not due the cell stress induced by the cytotoxicity.

**Fig 2:**
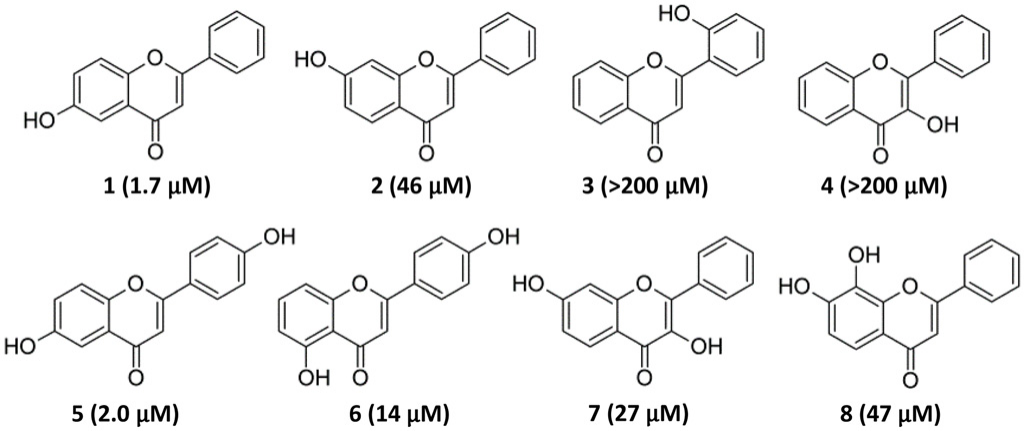
Structures of monohydroxylated and dihydroxylated flavones with IC_50_ against LPS-induced NO production.

On the basis of the inhibitory activity range of hydroxylated flavones, the potential structure-activity relationship was subsequently focused around the mono- and dihydroxyflavone scaffold as summarized in Fig. 2. Of the monohydroxylated flavones (**1**–**4**), the position of the hydroxyl group clearly played a critical role in the inhibitory activity, with 6-HO > 7-HO >> 3-HO and 2′-HO substitutions. On the other hand, the introduction of a second hydroxyl group on the 4′-position to 6-hydroxyflavone did not significantly impact the inhibitory activity. However, the shift of the hydroxyl group from the 6-position to the 5-position (flavone **5** vs **6** in Fig. 2) increased the IC_50_ by 7 fold. One hypothesis for the observed difference might be attributed to the resonance structure stability of benzopyrone core in flavones, which could be enhanced by electron-donating substituents. Structurally, the 6-position of the hydroxyl group in flavone **5** produced a stronger electron donating effect at the para-position to the oxygen atom of the benzopyrone ring to stabilize the resonance than the 5-position in flavone **6** at the meta-position to the oxygen atom. Unfortunately, other alternative positions for the dihydroxyl groups (flavones **7** and **8**) did not show a similar level of inhibitory activity as that of 6-hydroxy- or 4′,6-dihydroxyflavone. Furthermore, 2′,3,4′,6-tetrahydroxyflavone containing the 6-hydroxyl substitution had a high IC_50_ around 200 μM (Fig. 1). Therefore, our study suggested that 6-hydroxy- and 4′,6-dihydroxyflavones were a specialized subcategory of hydroxylated flavones for the inhibition of LPS-induced NO production in kidney mesangial cells.

Diadzein, a structural isomer of 4′,6-dihyroxyflavone was then investigated similarly to assess the importance of the flavone core to the anti-inflammatory activity. Structurally, the two hydroxyl groups of diadzein and 4′,6-dihyroxyflavone are located similarly at the extended para-positions of the core structure (Fig. 1). However, diadzein has the phenyl ring connected to the 3-position of benzopyrone whereas flavones have the phenyl ring connected to the 2-position. Strikingly, diadzein showed no significant inhibition of LPS-induced NO production up to 200 μM in contrast to the isomeric 4′,6-dihyroxyflavone with an IC_50_ of 2.0 μM. Diadzein could also be viewed as an isomeric analog of 7-hydroxyflavone (IC_50_ of 46 μM) if the hydroxyl group on the phenyl C-ring of diadzein was not considered. Thus, the low activity of diadzein versus those of similar hydroxylated flavones implied that the flavone core was an essential component for the activity against the LPS-induced NO production in addition to the position of the hydroxyl groups on the flavone structure.

### NO radical scavenging ability of selected hydroxylated flavones

**Fig 3:**
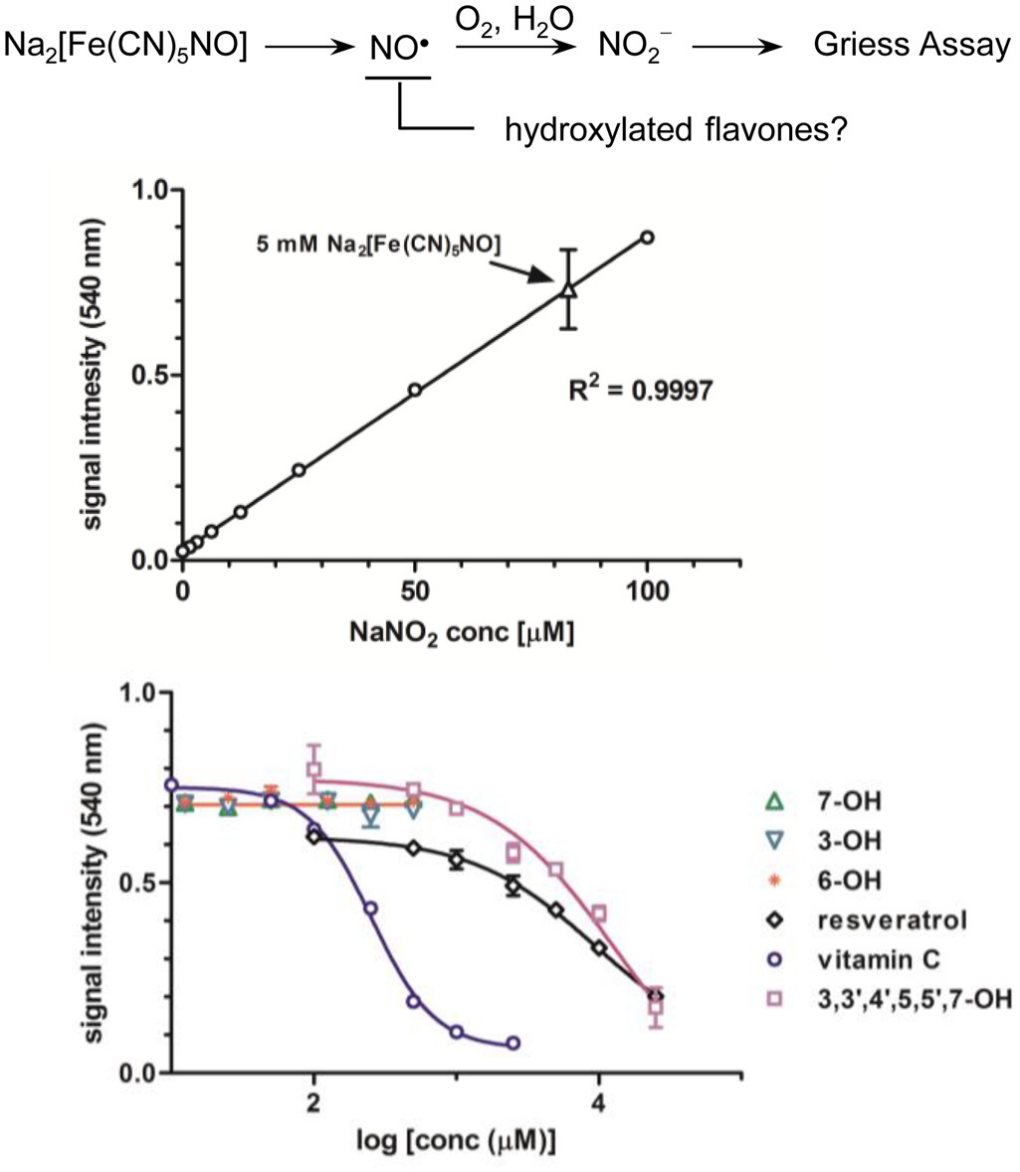
Direct NO radical quench study with selected monohydroxylated and polyhydroxylated flavones. Spontaneous generation of NO was achieved by a freshly prepared sodium nitroprusside stock solution (5 mM) in water in Griess assay (top). The direct quench of NO radicals were assessed on the 3-, 7- and 6-hydroxyflavone and 3,3′,4′,5,5′,7-hexahydroxyflavone with vitamin C and resveratrol as the positive controls.

One hypothesis for the high inhibitory activity of 6-hydroxyflavone was possibly due to the direct quenching of NO radicals by flavones in the LPS-induced cells or treatment media. Thus, flavones were investigated for their inhibition of the spontaneously generated NO radicals from sodium nitroprusside in solution [17]. We found that sodium nitroprusside at 5 mM produced a reasonable range of total nitrile concentration as measured by Griess assay as reported [17] (Fig. 3) that was effectively inhibited by ascorbic acid as the positive control with an IC_50_ of 254 μM. For hydroxylated flavones, none of the three monohydroxylated flavones (**1**, **2** or **4**) showed any activity up to 500 μM (Fig. 3), which was in contrast to the high inhibitory activity of **6** on the LPS-induced NO production. On the other hand, both resveratrol and 3,3′,4′,5,5′,7-hexahydroxyflavone (myricetin) had an IC_50_ of approximately 10 mM. These results implied that the high inhibitory activity on the LPS-induced NO production by 6-hydroxyflavone was not due to direct quenching of NO radicals while polyphenols such as myricetin did possess direct radical scavenging capability at the high concentration above 10 mM.

### 6-Methoxyflavone acts as a potent anti-inflammatory agent via inhibition of iNOS proteins in kidney mesangial cells

**Fig 4:**
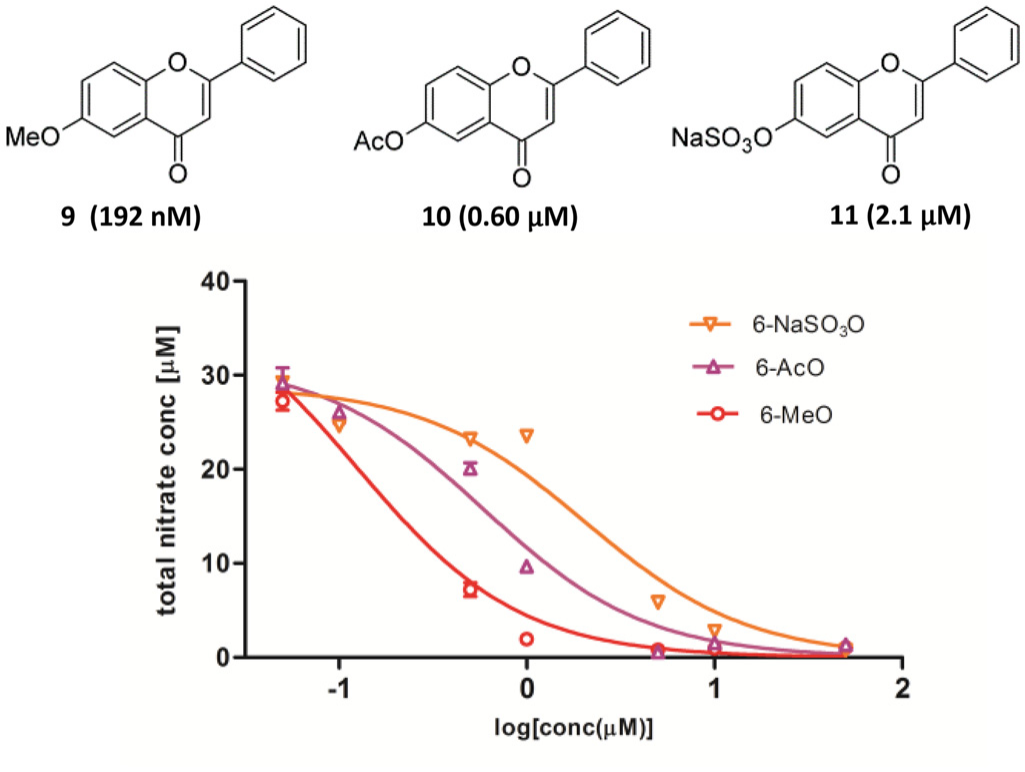
The inhibition of LPS-induced NO production in kidney mesangial cells by 6-methoxyflavone, 6-acetoxyflavone and flavone 6-sulfate. The total production of nitrite in the treatment media was measured with Griess assay at 48 h post 10 ng/mL LPS stimulation. IC_50_ values were obtained using the dose-response analysis provided by the Graphpad Prism software as shown in parenthesis.

Even though 6-hydroxyflavone exhibited a high activity against the LPS-induced inflammatory stimuli, the consequential idiosyncratic toxicity due to the noncompetitive inhibition of cytochrome P450 2C9 by 6-hydroxyflavone [18] could be a major concern for drug development. Thus, three alternative compounds with chemical modification of the 6-hydroxyl group including 6-methoxy- and 6-acetoxyflavones and flavone 6-sulfate were investigated (Fig. 4). Methylation of the 6-hydroxyl group could potentially weaken the hydrogen bond between the hydroxylated flavone and the Leucine 102 residue of P450 2C9 to decrease the non-competitive binding [19] while the acetylation as a pro-drug design might significantly change the pharmacokinetic profile. Flavone 6-sulfate is one of possible phase II metabolic products of 6-hydroxyflavone in the liver with sulfation of the phenolic group for subsequent renal clearance, and thus has a high potential for the anti-inflammatory application in kidney cells. For our study, 6-methoxyflavone is commercially available while 6-acetoxyflavone and flavone 6-sulfate were facilely synthesized in one step as described above in the Methods Section.

**Fig 5:**
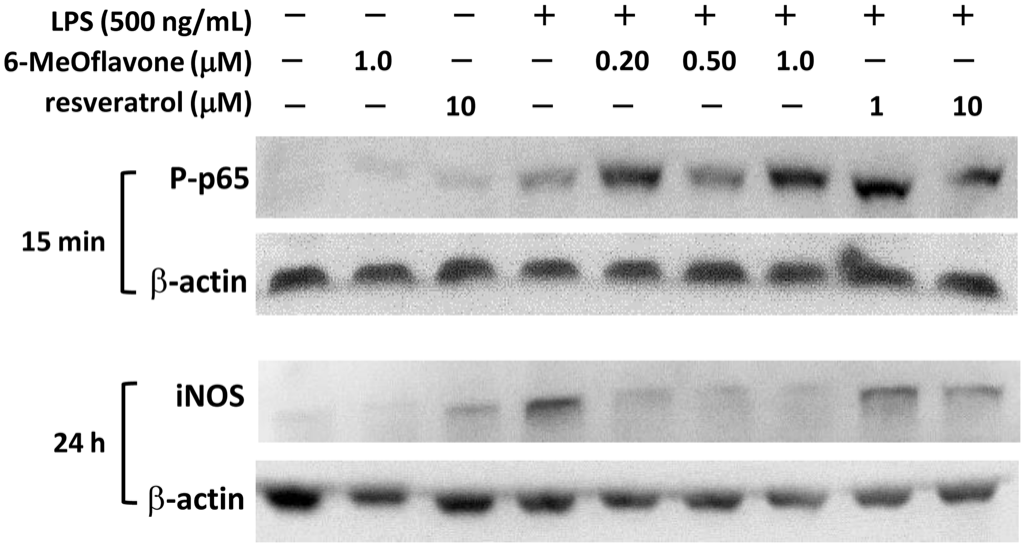
Western blot analysis of potential molecular targets by 6-methoxyflavone in kidney mesangial cells with LPS as the inflammatory stimuli. Cells were pretreated with 6-methoxyflavone or resveratrol for 12 h and then LPS was added. Cell lysate were collected after 15 min and 24 h incubation for activated NF-κB pathway and iNOS protein, respectively.

The inhibitory activity on the LPS-induced NO production by these modified 6-hydroxyflavones (**9–10**) was found to be impressively high with IC_50_ at 192 nM, 0.60 and 2.1 μM for 6-methoxyflavone, 6-acetoxyflavone and flavone 6-sulfate, respectively (Fig. 4). Specially, the activity of 6-methoxyflavone was almost 10-fold more potent than that of 6-hydroxyflavone (IC_50_ 1.7 μM). In addition, only weak cytotoxicity was observed on mesangial cells by 6-methoxyflavone at 1.0 μM, 6-acetoxyflavone at 10 μM and minimal for flavone 6-sulfate at 10 μM (see S1 Table). The underlying inhibitory mechanism was then focused on the LPS-stimulated cellular NF-κB pathway and 6-methoxyflavone due to its potency. Western blot analysis revealed that NF-κB pathway was activated upon the addition of LPS with significantly elevated levels of phosphorylated p65 protein (Fig. 5). Unfortunately, there was no observable inhibition of p65 activation by 6-methoxyflavone up to 1.0 μM. On the other hand, significant inhibition of the downstream inducible NO synthase (iNOS) upon LPS stimulation was consistently found with 6-methoxyflavone from 200 nM to 1.0 μM (Fig. 5). In addition, only weak inhibition was observed with the positive control resveratrol at 10 μM. Thus, these results suggested that the high activity of 6-methoxyflavone against the LPS-induced inflammatory stimuli was attributed to the potent inhibition of the downstream iNOS protein expression in kidney mesangial cells.

The high inhibitory potential of 6-hydroxyflavone in mesangial cells was unexpected because polyhydoxylated flavones such as quercetin and mericetin have been reported to be more effective anti-inflammatory flavones in macrophages and cancer cells [2,4]. Furthermore, the 10-fold increase on the activity of 6-methoxyflavone from 6-hydroxyflavone has not been similarly observed on methoxylated flavones on breast cancer cells [20]. On the other hand, methoxychalcones have been shown to exhibit significantly enhanced antimalarial activity as compared to hydroxylated chalcones due to an increased molecular refractory index of the methoxyl substituent [21], which might be a possible rationale for the enhanced activity of 6-methoxyflavone. While most of anti-inflammatory polyhydroxylated flavones have been shown to inhibit the phosphorylation of p65 in the NF-κB pathway [2,3], 6-methoxyflavone inhibited the downstream iNOS expression upon the inflammatory stimuli like LPS. All these results implied that 6-hydroxyflavone and 6-methoxyflavone were unique anti-inflammatory entities on kidney mesangial cells. In addition, 6-acetoxyflavone and flavone 6-sulfate may also be potentially effective in vivo as nephraprotective agents because the presence of sulfate group of flavone 6-sulfate and the prodrug design of 6-acetoxyflavone could possibly lead to an efficient delivery to the kidneys, which are currently under investigation. In conclusion, the identification of 6-hydroxyflavone and 6-methoxyflanvone with potent anti-inflammatory activity in kidney mesangial cells provides a new flavone scaffold and direction to develop potential naturally derived products for prevention and treatment of glomerolunephritis and glomerulosclerosis in diabetes.

## Supporting Information

S1 Table Viability of mesangial cells upon treatment with Group one flavones by MTT assay. ^a^MTT assay was carried out after the compound treatment for 48 h and cell viability was calculated as percentage relative to that of DMSO control. ^b^IC_50_ of compounds on the inhibition of the total nitrite production in the presence of 10 ng/mL LPS in mesangial cells after 48 h.
(PDF)

S2 Table Viability of mesangial cells upon treatment with Group two and three flavones by MTT assay. ^a^MTT assay was carried out after the compound treatment for 48 h and cell viability was calculated as percentage relative to that of DMSO control. ^b^IC_50_ of compounds on the inhibition of the total nitrite production in the presence of 10 ng/mL LPS in mesangial cells after 48 h.
(PDF)

S3 Table Viability of mesangial cells upon treatment with Group four and five flavones by MTT assay. ^a^MTT assay was carried out after compound treatment for 48 h and cell viability was calculated as percentage relative to that of DMSO control.
(PDF)
