# Supplementary material for: 6-Hydroxyflavone and Derivatives Exhibit Potent Anti-Inflammatory Activity among Mono-, Di- and Polyhydroxylated Flavones in Kidney Mesangial Cells
Source: PLoS One. 2015 Mar 19;10(3):e0116409. doi: 10.1371/journal.pone.0116409 (PMC4366162; doi:10.1371/journal.pone.0116409)
Supplement: S1 Table — aMTT assay was carried out after the compound treatment for 48 h and cell viability was calculated as percentage relative to that of DMSO control. bIC50 of compounds on the inhibition of the total nitrite production in the presence of 10 ng/mL LPS in mesangial cells after 48 h. (PDF) [file pone.0116409.s001.pdf]

compounds	6-MeO (192 nM)[b]	6-AcO (0.60 μM)[b]	6-HO (1.7 μM)[b]	4′,6-HO (2.0 μM)[b]	6-NaSO$_3$O (2.1 μM)[b]
0.10 μM	109 ± 2 %	107 ± 3 %	108 ± 5 %	98 ± 3 %	100 ± 2 %
1.0 μM	83 ± 2 %	105 ± 2 %	117 ± 4 %	105 ± 1 %	106 ± 2 %
10 μM	68 ± 1 %	89 ± 2 %	116 ± 3 %	115 ± 5 %	107 ± 4 %

.
