# Supplementary material for: 6-Hydroxyflavone and Derivatives Exhibit Potent Anti-Inflammatory Activity among Mono-, Di- and Polyhydroxylated Flavones in Kidney Mesangial Cells
Source: PLoS One. 2015 Mar 19;10(3):e0116409. doi: 10.1371/journal.pone.0116409 (PMC4366162; doi:10.1371/journal.pone.0116409)
Supplement: S2 Table — aMTT assay was carried out after the compound treatment for 48 h and cell viability was calculated as percentage relative to that of DMSO control. bIC50 of compounds on the inhibition of the total nitrite production in the presence of 10 ng/mL LPS in mesangial cells after 48 h. (PDF) [file pone.0116409.s002.pdf]

compounds	3,3′,7-HO (11 µM)[b]	3,3′,4,7-HO (11 µM)[b]	resveratrol (11 µM)[b]	4′,5-HO (14 µM)[b]	3′,4′,5′,7-HO (15 µM)[b]	3,3′,4′,5,7-HO (22 µM)[b]	3,7-HO (27 µM)[b]	7,8-HO (47 µM)[b]	7-HO (46 µM)[b]
5 µM	109 ± 1 %	101 ± 2 %	106 ± 2 %	106 ± 2 %	96 ± 1 %	-	-	-	-
10 µM	108 ± 2 %	99 ± 1 %	104 ± 2 %	101 ± 4 %	92 ± 2 %	94 ± 2 %	101 ± 1 %	100 ± 2 %	94 ± 3 %
50 µM	86 ± 1 %	95 ± 4 %	106 ± 1 %	89 ± 6 %	81 ± 3 %	80 ± 5 %	119 ± 7 %	88 ± 3 %	93 ± 4 %
100 µM	-	-	-	-	-	78 ± 1 %	74 ± 1 %	89 ± 3 %	79 ± 4 %
