# Supplementary material for: 6-Hydroxyflavone and Derivatives Exhibit Potent Anti-Inflammatory Activity among Mono-, Di- and Polyhydroxylated Flavones in Kidney Mesangial Cells
Source: PLoS One. 2015 Mar 19;10(3):e0116409. doi: 10.1371/journal.pone.0116409 (PMC4366162; doi:10.1371/journal.pone.0116409)
Supplement: S3 Table — aMTT assay was carried out after compound treatment for 48 h and cell viability was calculated as percentage relative to that of DMSO control. (PDF) [file pone.0116409.s003.pdf]

compounds	100 μM	200 μM
2′,3,4′,5,7-HO	111 ± 4 %	105 ± 6 %
3′,4′,5′,5,7-HO	86 ± 3 %	107 ± 4 %
2′,3,4′,6-HO	114 ± 1 %	108 ± 1 %
2′,3,7,8-HO	97 ± 1 %	95 ± 1 %
3,3′,4′,5,7,8-HO	78 ± 2 %	81 ± 5 %
2′-HO	106 ± 5 %	103 ± 3 %
3-HO	93 ± 2 %	104 ± 12 %
4′,5,7-HO	95 ± 5 %	88 ± 4 %
3,3′,4′,5′,5,7-HO	78 ± 1 %	81 ± 3 %
Daidzein	92 ± 2 %	86 ± 5 %
